# The impact of urban green space on the health of middle-aged and older adults

**DOI:** 10.3389/fpubh.2023.1244477

**Published:** 2023-10-10

**Authors:** Qiangyi Li, Yangqing Liu, Lan Yang, Jiexiao Ge, Xiaona Chang, Xiaohui Zhang

**Affiliations:** ^1^School of Economics and Management, Guangxi Normal University, Guilin, China; ^2^Development Institute of Zhujiang-Xijiang Economic Zone, Guangxi Normal University, Guilin, China; ^3^Economics and Management School, Wuhan University, Wuhan, China

**Keywords:** urban green space, physical health, mental health, mechanism analysis, middle-aged and older adults

## Abstract

**Introduction:**

Urban green space is one of the most closely related ecosystem services to residents’ lives, and it can be regarded as a preventive public health measure. Residents living in parks and other green environments can help improve their physical and mental health, reduce stress and even prevent crime and violence. Therefore, based on the actual situation in China, this paper analyzes the relationship between urban green space and the health of middle-aged and older adults and its mechanisms.

**Methods:**

This study used multiple linear regression, based the data from the China Health and Retirement Longitudinal Study (CHARLS) in 2013, 2015, and 2018, to explore the relationship between urban green space and the health of middle-aged and older adults. At the same time, group regression was conducted to identify the heterogeneity of health effects of urban green space.

**Results:**

The research shows that the increase of urban green space areas can significantly improve the health status of middle-aged and older adults. After a series of robustness tests, the results are still valid. In addition, the health effects of urban green space are different because of gender, age, education level, marital status residence, geographical location of the respondents and park quantity distribution. Further research found that reducing hot weather and optimizing air quality are the potential mechanisms of urban green space affecting the health of middle-aged and older adults, providing new evidence for the causal mechanism between urban green space and the health of middle-aged and older adults.

**Discussion:**

This study expanded the research scope of the impact of urban green space on the health of middle-aged and older adults, covering a representative sample in China. The results show that urban green space has an important impact on the health of middle-aged and older adults. Policy suggestions are made to help cities optimize the landscape and residents to enjoy ecology.

## 1. Introduction

Many countries around the world are facing the severe challenge of an aging population (1–3). According to United Nations data, by 2030, one-sixth of the people in the world will be over 60 years old. From 2020 to 2030, the population over 60 years old will increase from 1 billion to 1.4 billion. By 2050, the world’s population over 60 years old will double to 2.1 billion. Between 2020 and 2050, the number of people over the age of 80 is expected to triple to 426 million. Similarly, the degree of population aging in China will continue to deepen rapidly, and the dilemma of “getting old before getting rich” is still challenging to break through (4). According to the “2021 National Bulletin on the Development of Ageing” issued by the China Municipal Health and Wellness Commission and the National Office for Ageing, by the end of 2021, there had been 267.36 million older adults aged 60 and above in China, accounting for 18.9% of the total population. There are 200.56 million older adults aged 65 and above in China, accounting for 14.2% of the total population. In order to actively respond to the concept of aging, China issued the Outline of Healthy China 2030, and the report of the 20th National Congress of the Communist Party of China further proposed to implement the strategy of actively responding to population aging and promoting the construction of healthy China. Protecting the physical and mental health of middle-aged and older adults and improving the quality of life of middle-aged and older adults is the meaning of the healthy China, and it is also an important measure to practice the people-oriented concept. However, due to aging, the physical and mental condition of middle-aged and older adults has changed obviously, and the possibility of suffering from diseases (such as chronic diseases and dementia) has increased (5). In addition to physical disorders, the frequency of mental illness among the older adults is also increasing (6, 7), depression, anxiety and other psychological problems are a significant burden for older adults around the world (8, 9). Mental illness has also been identified as the leading cause of non-fatal health loss in the world, and health problems caused by negative psychology, including self-harm, dementia and suicide, are also widespread, bringing heavy burdens to families and society. Promoting the physical and mental health of the enormous middle-aged and older groups is a position that cannot be ignored (10, 11).

In the process of aging, the optimization of social environment conditions is the key to improve the physical and mental health of older adults and promote the development of active aging. Urban green space not only beautifies the urban environment but also is the principal public place where urban residents live, which has a subtle influence on the lifestyle of residents (12, 13). In 1998, in the Basic Terminology Standard of Urban Planning, urban green space was defined as “green land specially used to improve the ecology, protect the environment, provide recreation places for residents and beautify the landscape.” In 2002, the Classification Standard of Urban Green Space expanded the definition of urban green space and defined green space as “a kind of urban land with vegetation as the main existing form, which is used to improve urban ecology, protect the environment, and provide recreation places for residents and beautify the city.” The utilization of urban green space not only includes the planning of space and function but also needs to pay attention to its role in the urban ecological environment, providing leisure and entertainment places and beautifying the city so as to create a comfortable living environment for the public. As an important part of the city, urban park green space has an important impact on the health of middle-aged and older adults. Regular physical activities in green spaces can indirectly or directly benefit the physical and mental health and happiness of residents (14, 15), and the benefits to the older adults are more obvious (16). This is because green spaces help residents get more exercise and relax. This significantly reduces not only cardiovascular disease, but also the health risks of respiratory disease, high blood pressure, paralysis, diabetes and other chronic diseases. A large number of studies have shown that urban green space has a health promotion function and can provide people with a series of ecosystem services and universal health benefits (17).

## 2. Literature review and theoretical mechanism

### 2.1. Literature review

The existing literature mainly focuses on the following aspects in discussing urban green space and the health of the older adults. Firstly, from the perspective of the improvement effect of urban green space on the residents’ environment. Many studies believe that urban green space provides residents with good conditions for outdoor sports, and green space in different regions has diverse functions and provides health benefits for residents from different angles: urban green space is an outdoor space covered by green vegetation in urban areas, such as forests, parks, grasslands, green belts, river wetlands, etc. Green vegetation has a good air purification function, which can directly play the role of intercepting pollution particles through retention and attachment, and can also be realized by changing climate factors such as wind fields (18). Therefore, urban parks and green spaces can improve the ecological environment, reduce the level of urban pollution control, improve residents’ quality of life, and thus improve their subjective well-being (19). Square green space can provide fitness venues, improve urban residents’ sports and outdoor activities, improve residents’ willingness and ability to exercise, strengthen their physique, and improve their physical function (20, 21). And outdoor social communication can improve individual emotional enthusiasm, enhance social cohesion, and campus residential green space can beautify the living environment.

Secondly, urban green space can also play a positive psychological healing effect. More and more evidence shows that outdoor activities are positively related to adults’ mental health, and more contacting with green space can reduce people’s anxiety and depression (22). More research results show that urban green vegetation can reduce urban noise pollution (23), so it is speculated that urban traffic noise is the chief culprit affecting people’s mental health. Traffic noise will have adverse effects on children’s neurological development through stress, worry and sleep quality (24).

Finally, from the existing research methods on the two, some scholars have quantified and drawn the urban green space by constructing a spatial model and analyzing the critical factors that affect people’s health based on the GIS model and spatial data (25). Researchers use the CLM model developed in 2016 to predict neuropsychological data accurately, and then fully measure the design quality of urban green space (26).

The existing research has explored the relationship between urban green space and human health, providing us with rich perspectives and profound insights, but there may be three areas for improvement. First, the existing research is more based on a specific city or region, based on manual survey data, to explore the impact of specific parks on the health of surrounding residents, which may not be universal. Secondly, the way of describing human health in existing articles is based on the incidence of a specific disease, or the individual emotional score is obtained by answering questions. Few kinds of literature comprehensively examine many aspects of human health and comprehensively describe the health level by combining the psychological and physical dimensions of the human body. Thirdly, most of the literature has empirically tested the relationship between urban green space and the health of the older adults but failed to explore the causal mechanism behind the relationship further.

Compared with previous studies, this study may have the following three contributions. First, the existing research focuses more on the influence of air pollution, extreme weather, urban development and community activities on the health of the older adults, and less literature puts urban green space and the health of the older adults into a unified analysis framework, which provides a new academic perspective for studying the health of the older adults. Secondly, the physical health status of individual samples is obtained by combining the incidence of acute and chronic diseases. Besides, the individual mental health status is obtained using the Depression Scale (CESD), and the individual depression emotion value is calculated, which can reflect the individual’s physical and mental health level more comprehensively from the two latitudes of psychology and body. Thirdly, the study attempts to explain the internal mechanism behind the positive impact of urban green space on the health of the older adults from the perspective of improving urban high-temperature weather and urban air pollution and provide a richer perspective for related research.

Based on the above problems, this study uses the data from the China Health and Retirement Longitudinal Study (CHARLS) to analyze the impact of urban green space on the health of middle-aged and older adults. First of all, according to the acute and chronic diseases, eight adverse emotional problems, and two positive emotional problems in the questionnaire, the health status of residents is quantified in detail, and the health of middle-aged and older adults is divided into two dimensions: physical health and mental health. Secondly, the influence of urban green space on the physical and psychological health of middle-aged and older adults is empirically tested by multiple linear regression, and a series of robustness tests are also carried out to support the empirical results. And further, we identify the internal relationship between urban green space and middle-aged and older adults’ physical and mental health. Finally, the heterogeneity is discussed from gender, age, and place of residence.

The possible marginal contributions of this study are as follows: First, different from the previous general and single indicators, this paper turns to a more specific and multi-dimensional measurement, measuring the health level of middle-aged and older adults from two dimensions of physical health and mental health, and more accurately analyzing the health effects of urban green space on middle-aged and older adults. Secondly, analyze and test the mechanism channels of urban green space affecting the physical health and mental health of older adults, supplement the existing research literature at the level of mechanism analysis, and explain the internal relationship between urban green space and the health of middle-aged and older adults from the perspectives of economics, environmentalism, and sociology. Thirdly, because of individual differentiation, a series of heterogeneity analyses, such as gender and place of residence are carried out. A more detailed classification discussion is carried out at the individual level so as to make suggestions on the integration and optimization of urban green space layout in the future and the guarantee mechanism of the health level of different older groups.

### 2.2. Theoretical mechanism

With the increasing urbanization of the world and the reduction of the relationship between man and nature, the impact of urban green space on human health has attracted more and more attention from academic circles. During the COVID-19 pandemic, that is, during the implementation of the social distance policy, restrictions on mobility, concerns about safety, and restrictions on access, it tended to use urban green space (UGS) to provide alternative space for social interaction and health (27). Through an online survey conducted from March to April, 2021, it was found that respondents will continue to use UGS during the pandemic and think it is more beneficial to health (28–31). Most studies have found that there is an overall positive correlation between green space and health (32, 33). Further, some scholars have found that normalized vegetation index (NDVI), vegetation coverage, park coverage and street lawn are positively correlated with mental health of the older adults (34–36). Recent research shows that being close to nature (such as parks, green spaces, etc.) can reduce the stressors related to loneliness and reduce the stress level (37–39). At the same time, some studies have proved that urban greening has a protective effect on cardiovascular risk factors, diseases and mortality (40, 41). Urban green space is a crucial resource to effectively improve urban environment and human health. More and more evidence shows their positive effects on health and wellbeing (42–44). Therefore, Hypothesis 1 is put forward.

*Hypothesis 1:* Increasing urban green areas can better improve the health status of middle-aged and older adults.

High ambient temperature is related to many health effects, including premature death. The combination of global warming caused by climate change and the expansion of the global built environment means that urban heat island (UHI) is expected to intensify, accompanied by adverse effects on population health (45, 46). A large number of studies show that urban green space can effectively alleviate the heat island effect. By increasing the coverage area of green space, the urban thermal stress and global warming potential gas can be reduced to protect the environment to reduce the negative impact on people’s health (47–50). Massaro et al. (51) based on the spatial regression model of remote sensing data, evaluated the extreme exposure of people to high temperature (LST) in the urban environment of 200 cities according to surface characteristics such as vegetation coverage and distance from water bodies. The research results showed that urban vegetation played a considerable role in reducing the exposure of the urban population to extremely high temperatures (LST). Therefore, Hypothesis 2 is put forward.

*Hypothesis 2:* Urban green space can improve the health status of middle-aged and older adults by reducing the temperature value of sweltering weather.

By using the data from the large-scale survey of “the world’s first-class harmonious and livable capital” in Beijing in 2018 to investigate the influence of subjective and objective characteristics of UGS on residents’ self-rated health (SRH) and applying binary Logistic regression model. It is found that social interaction and air quality perception are the two main mediating variables that UGS affects residents’ self-rated health (52). At the same time, some scholars further pointed out that the content of PM_2.5_ in the air can be reduced by increasing the investment in urban green space. Particulate matter (PM_2.5_ and PM_10_) is an essential source of urban air pollution and poses a severe threat to the health of urban residents (53). Urban vegetation has a specific ecological function of reducing atmospheric particles and is vital in improving the urban atmospheric environment. Based on hourly measured data of particulate matter concentration and high-resolution remote sensing images, some scholars estimated the total reduction and removal rate of PM_2.5_ and PM_10_ in the atmosphere by urban green space within the Fifth Ring Road in Beijing. It has been found that urban green space has a significant reduction effect on PM_2.5_ and PM_10_ (54, 55). Some scholars take different types of green space as the research object and test the air ion concentration, PM_2.5_ concentration, temperature, humidity, wind speed, and other indicators, and analyze the temporal and spatial characteristics of air harmful ion concentration and PM_2.5_ concentration in different types of green space. The research shows that the concentration of atmospheric particles presents other trends throughout the day, and small particles (PM_2.5_) offer a “low morning and evening, high noon” trend, while large particles (PM_10_ and TSP) follow the trend (56). Therefore, Hypothesis 3 is put forward.

*Hypothesis 3*: Urban green space can improve the health level of middle-aged and older adults by improving air quality.

## 3. Materials and methods

### 3.1. Regional characteristics

China is located on the west coast of East Asia and the Pacific Ocean, with a land area of about 9.6 million square kilometers and more than 18,000 kilometers of coastline. China has highly complicated geographical and ecological characteristics. This area has forests, shrubs, alpine vegetation, and aquatic and other vegetation types. It is one of the largest biodiversity countries in the world and has the second wealthiest plant population (57). There are tropical forests and seasonal rainforests in the eastern monsoon region, subtropical evergreen broad-leaved forest. Deciduous broad-leaved forest in warm temperate zone. And coniferous and broad-leaved mixed forests in temperate zone. There are coniferous forests in the cold zone. Regional vegetation spans a wide range of latitude and longitude (58), including unique plateau vegetation and altitude spectrum of several peaks on the Qinghai-Tibet Plateau (59). According to the data of the World Bank, the proportion of forest area in China increased from 18.85 to 23.43% from 2000 to 2020 and increased year by year during the inspection period, with a growth rate of 24.30%, which is relatively high. The proportion of forest area in the world is about 31% in 20 years, about 10% higher than that in China. The proportion of forest area in developed countries has remained unchanged for many years, with the United States at about 33%, Britain at about 12%, and Japan at about 68%, which has been in a reasonably high proportion range worldwide. Generally speaking, the green forest area is relatively large, but the proportion of green forest area still has room for improvement compared with developed countries in China.

Due to the geographical and climatic advantages, China’s parks and green spaces have developed well. The China Municipal Government is committed to improving the urban ecological environment, hoping to beautify the urban living environment and provide recreational places for residents by expanding urban green spaces. In recent 10 years, the area of urban green space in China has obviously expanded with the efforts of the China Municipal Government.

**Figure 1:**
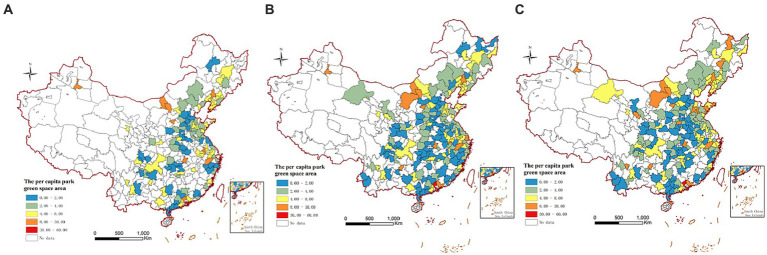
Per capita green area of China city. **(A)** 2013; **(B)** 2015; **(C)** 2018. The standard map production is based on the standard map service website of the Ministry of Natural Resources, with the approval number of GS (2019) No.1822. And, no changes to the base map boundary.

In this study, the samples of 221 prefecture-level cities in China are selected as the objects of investigation. Figure 1 shows each selected city sample’s per capita green area in 2013, 2015, and 2018. From the time trend, the number of residential green areas in cities in China is increasing, and the number of cities in the range of 2–4 square meters is increasing, too. Specifically, in 2013, there were many deficiencies in the samples, and the urban per capita green area was mainly within the range of 0–8 square meters, and the residential green area in the eastern coastal areas was higher than that in the central and western regions, and the per capita green area in a few northern cities reached the range of 8–30 In 2015, cities’ per capita green area increased significantly compared with that of 2013. The number of cities in the 0–2 square meters range increased significantly, and those in the 4–8 square meters increased. The per capita green area of very few cities reached 8–30 square meters. In 2018, the number of investigation samples has increased significantly, and more cities have entered the range of about 10 square meters. What is gratifying to us is that the number of cities in the range of 2–4 square meters has reached the highest in the three investigation years.

### 3.2. Demographic characteristics

According to the sixth national population census, 83.15% of the older adults consider themselves healthy and basically healthy, and 16.85% consider themselves in poor health. In 2015, the fourth survey on the living conditions of the older adults in urban and rural areas of China showed that 75.24% considered themselves to be healthy or average, and 24.76% considered themselves to be in poor or very poor health. According to the Health China Action Promotion Committee, in 2018, about 8.3 older adults lived with diseases in China, and there were 180 million older adults with one or more chronic diseases, accounting for 75%. China’s implementation of the strategy of actively responding to population aging has entered the fast lane. More and more studies have realized that healthy aging is the only way for China to actively cope with the aging population, and solving health problems can basically resolve the negative effects of aging.

Micro-individual data in this paper come from 2013, 2015, and 2018 follow-up surveys of China Health and Retirement Longitudinal Study (CHARLS data source: https://charls.charlsdata.com/), which is a national sampling survey project sponsored by the National Development Research Institute of Peking University. CHARLS aims to collect a set of high-quality micro-data representing the families and individuals of middle-aged and older adults aged 45 and above in China to analyze the problem of population aging in China and promote interdisciplinary research on aging. CHARLS data is collected every 2–3 years. The baseline survey in 2011–2012 covered 28 provinces, 150 counties, and 450 villages (communities) in China, involving 17,708 people from 10,257 families. Three follow-up surveys were conducted in 2013, 2015, and 2018. The study mainly includes essential personal characteristics, health status and function, pension, family income, and other information. By the time the national follow-up visit was completed in 2018, the sample had covered 19,000 respondents from 12,400 households, which can fully reflect the overall situation of middle-aged and older adults in China. At present, it has been widely used in residents’ economic, social, and health research.

### 3.3. Data processing

Considering the lack of cities in the initial stage of inclusion and the problem of matching with CHARLS, this paper selects the data of urban green space, air quality, and high-temperature weather from 2013 to 2018 in the yearbook for analysis and according to the city code and year, the China Urban Construction Statistical Yearbook was merged with the CHARLS database. Then the missing cases were deleted by state, and finally, the micro-individual samples covering 221 cities (autonomous) regions were matched.

### 3.4. Measurement and selection of indicators

#### 3.4.1. Dependent variables

Based on referring related research (60), the health status of middle-aged and older adults is measured from two dimensions: physical health and mental health, including physical illness and depression. Physical diseases are characterized by the total number of chronic diseases: “digestive diseases, kidney diseases, lung diseases, liver diseases, arthritis, asthma, diabetes, dyslipidemia, hypertensive neurological diseases” and acute diseases: “heart disease, stroke, cancer” in the CHARLS questionnaire. Each condition counts as one point, and the higher the score, the worse the physical health. Depressed mood was used to evaluate the mental health of the interviewees by designing eight adverse emotional problems and two positive emotional problems according to the depression scale (CES-D) in the CHARLS questionnaire and their feelings and behaviors last week. The issues cover (1) I am troubled by some trivial matters. (2) I find it difficult to concentrate on my work. (3) I feel depressed. (4) I find it hard to do anything. (5) I feel scared. (6) I feel lonely. (7) I do not sleep well. (8) I do not think I can continue my life. (9) I am full of hope for the future. (10) I am delighted. Calculating the score according to the respondents’ answers, and the higher the score gets, the worse the mental health is.

#### 3.4.2. Independent variables

Urban green space is the independent variable of this study, and the per capita park green space area is selected to represent it.

#### 3.4.3. Control variables

Because residents’ health will be influenced by individual characteristics, family characteristics, and living habits, this study draws lessons from previous studies on a health level. It introduces 12 indicators, including age, gender, marriage, smoking, family income, expenditure, and whether toilet facilities are available, etc. as control variables. The meanings and descriptive statistics of the main variables are shown in Table 1.

**Table 1:** Meaning and descriptive statistics of main variables.

Variables	Symbol	Definition	Meaning and assignment of variables	Mean	SD
Dependent variables	*PHS*	Physical illness	0→13, the higher the score, the worse the physical health	1.6792	1.6823
	*DEP*	Depressive mood	0→30, the higher the score, the worse the mental health	7.7371	6.1276
	*BOD*	Body function	6→24, the higher the score, the worse the body motor function	8.2493	3.2691
Independent variables	*GRE*	Per capita Park green area square meters/person	Total Park green area/total population	3.5599	3.8095
	*COV*	Green coverage rate of built-up area	Green coverage area/urban built-up area *100%	40.7080	5.6955
Control variables	*AGE*	Age	The age of the interviewee in that year (years old)	61.0612	10.1041
	*GEN*	Gender	Male = 0; Female = 1	0.5208	0.4996
	*MAR*	Marriage	Cohabitation, married = 1; Unmarried, separated, divorced, widowed =0	0.8631	0.3438
	*REG*	Town and country	Living in rural areas =1, urban =0	0.5884	0.4921
	*EDU*	Degree of education	1→10, the higher the score, the higher the education level	3.4086	1.8759
	*SLE*	Average daily sleep time last month	Average daily sleep time in last month (h)	6.3157	1.8930
	*HEA*	Childhood health	1→5, the higher the score, the worse the physical health	2.6835	1.1155
	*DRI*	Frequency of drinking last year	0→9, the higher the score, the higher the frequency	1.6281	2.7451
	*SMO*	Smoke	Smoking = 1; No smoking = 0	0.4273	0.4947
	*CHI*	Number of children	Number of children raised by families (number)	2.6129	1.3987
	*EXP*	Household expenditure	The total amount of household expenditure is logarithm	12.6033	5.7577
	*TOI*	Use toilet equipment	Yes = 1; No = 0	0.0204	0.1415
Mechanism variables	*PM_2.5_*	Air quality	Annual average value of PM_2.5_	47.6436	17.6996
	*TEM*	Highest temperature	The annual extreme maximum temperature is Celsius	38.0509	1.5722

#### 3.4.4. Mechanism variables

At present, there are few kinds of literature about the channels through which urban green space affects the health of middle-aged and older adults. Based on the above mechanism, extreme temperatures become more frequent, intense, and common under climate change, which may affect individuals’ physical and mental health and cause negative emotions such as anxiety and psychological stress (61). The research will focus on the micro-mechanism of urban green space’s impact on middle-aged and older adults’ health from extremely high-temperature weather and air quality changes. Because urban green space helps to reduce the surface temperature and reduce the heat island effect. This can prevent residents from being in high-temperature environments for a long time and reduce the risk of heatstroke and other diseases related to high temperatures. Therefore, the annual extreme maximum temperature of Celsius is selected as a measure index to measure high-temperature weather, and the influence mechanism of urban green space on the health status of middle-aged and older adults is discussed. In addition, urban green space can effectively absorb pollutants in the air, reduce suspended particles in the air and improve air quality, which is conducive to reducing the risk of respiratory diseases for residents. Therefore, the average concentration of PM_2.5_ is selected as an index to measure the air quality, and the influence mechanism of urban green space on the health of middle-aged and older adults is measured.

## 4. Research design

### 4.1. Construction of regression model

The regression model of middle-aged and older adults’ health and urban green space was built by stata16 software. Because the scores of provincial diseases and depression were continuous variables, multiple linear regression was selected to analyze the impact of urban green space on middle-aged and older adults’ health. This paper focuses on the direct relationship between urban green space and middle-aged and older adults’ health level. In order to more accurately study this direct causal relationship, the following measurement model is constructed to verify:


(1) $$Y_{ict}=\alpha +\beta {GRE}_{ct}+\delta X_{ict}^{\prime }+\varepsilon_{\mathrm{it}}$$

Among them, *i* means the respondent, *c* means the city where the respondent is located, and *t* represents the year. $Y_{ict}$ represent the mental health and physical health variables at the individual level, and ${GRE}_{ct}$ means the urban green space variables in the city where the interviewee is located in, $X_{ict}^{\prime }$ indicates the control variables that may affect the health level. And, $\varepsilon_{\mathrm{it}}$ is a random error term.

## 5. Analysis of empirical results

### 5.1. Benchmark regression results

The effects of urban green space on middle-aged and older adults’ physical and mental health were investigated by regression method. The dependent variables in columns (1)–(4) of Table 2 are the physical health and mental health of middle-aged and older adults, respectively. In order to reduce the estimation bias caused by missing variables, we control the individual characteristics and family characteristics of middle-aged and older adults, respectively. Among them, The columns (1) and (3) indicate the control of individual level control variables, and columns (2) and (4) indicate the control of individual and family level control variables. It can be found that the estimation coefficients are −0.0167, −0.0171, −0.0657, and −0.0623, respectively, and all of them have passed at least 5% significance level test, indicating that the increase of per capita green park space will significantly reduce the scores of physical diseases and depression, that is, urban green space can effectively improve the physical health and mental health of middle-aged and older adults.

**Table 2:** Benchmark regression results.

Variables	(1)	(2)	(3)	(4)
	PHS	PHS	DEP	DEP
GRE	−0.0167***	−0.0171***	−0.0657**	−0.0623***
	(−3.4646)	(−3.2282)	(−2.4634)	(−2.6456)
Individual controls	YES	YES	YES	YES
Family controls	NO	YES	NO	YES
_cons	−1.0708***	−1.0002***	12.1694***	12.4092***
	(−6.4151)	(−4.8897)	(22.9142)	(20.1335)
*N*	25,256	25,210	24,932	24,892
*R* ^2^	0.1221	0.1259	0.1359	0.1385

*T* value is indicated in brackets. *, **, *** represent the significance levels of 10%, 5% and 1%, respectively.

### 5.2. Robust test

#### 5.2.1. Replacing independent variable

In order to increase the reliability of the research conclusion, the green coverage rate of the built-up area is used to reflect the urban green space level of the city (district) under its jurisdiction, and the specific results are shown in columns (1)–(4) of Table 3. It can be seen that the estimation coefficients are all negative, and the green coverage rate of the built-up area substantially reduces the physical diseases of the older adults, indicating that the improvement of the green coverage rate of the built-up area will significantly improve the health status of middle-aged and older adults. The robustness of the benchmark regression mentioned above is proved. When the individual and family control variables are not added, the influence of urban green coverage on the depression of older adults is significantly harmful. In contrast, the improvement of urban green range on the depression of more senior people needs to be further verified after adding various control variables.

**Table 3:** Replace the independent variable for robustness test.

Variables	(1)	(2)	(3)	(4)
	PHS	PHS	DEP	DEP
COV	−0.0111**	−0.0117**	−0.0418**	−0.0408**
	(−2.2582)	(−2.3479)	(−1.9963)	(−2.0086)
Individual controls	YES	YES	YES	YES
Family controls	NO	YES	NO	YES
_cons	−0.6791**	−0.6037**	13.6373***	13.7860***
	(−2.4374)	(−2.0283)	(13.9488)	(13.4559)
*N*	25,259	25,213	24,935	24,895
*R* ^2^	0.1221	0.1260	0.1358	0.1385

*T* value is indicated in brackets. *, **, *** represent the significance levels of 10%, 5% and 1%, respectively.

#### 5.2.2. Replacing dependent variable

In order to increase the rigor of the research conclusion, this paper measures the health status of middle-aged and older adults with physical function indicators, covering the following six problems: it is difficult to lift or carry heavy objects weighing more than 10 kg, it is challenging to reach arms above shoulders, it is difficult to bend over, kneel or squat down, it is tough to climb several stairs without rest, it is difficult to stand up from a chair after sitting for a long time, and it is difficult to pick up a small coin from a table. Respondents answered “I do not have any difficulties” = 1; “It’s a little difficult but it can be done” = 2; “I am in trouble and need help” = 3; “I cannot do it” = 4, and the scores of 6 questions add up to the physical function score. The higher the score gets, the worse the health status is. As the score of physical function is a continuous variable, a multiple linear regression model is used to estimate it, and the specific results are shown in columns (1)–(3) of Table 4. It can be seen that the estimation coefficients are all significantly negative at the level of 1%, indicating that the increase of per capita park green area substantially reduces the score of physical function. That is, urban green space effectively improves the health status of middle-aged and older adults, which proves the robustness of the previous benchmark conclusions.

**Table 4:** Replace the dependent variable for robustness test.

Variables	(1)	(2)	(3)
	BOD	BOD	BOD
GRE	−0.0344***	−0.0197***	−0.0173***
	(−3.0395)	(−3.2100)	(−2.9716)
Individual controls	NO	YES	YES
Family controls	NO	NO	YES
_cons	8.0992***	4.2695***	4.7250***
	(99.5053)	(16.4364)	(17.6046)
*N*	33,013	24,597	24,555
*R* ^2^	0.0064	0.1811	0.2116

*T* value is indicated in brackets. *, **, *** represent the significance levels of 10%, 5% and 1%, respectively.

#### 5.2.3. Shorten the time window

In order to further test the robustness of the benchmark conclusion, the number of samples in 2013 and 2015 was excluded, and the newer time data, namely the sample data in 2018, was selected for the robustness test. The results are shown in Table 5. After controlling the variables of individual and family characteristics, respectively, the estimation coefficient is significantly negative, and the increase of per capita park green area substantially reduces the scores of physical illness and depression, which is consistent with the benchmark conclusion and passes the robustness test.

**Table 5:** Shorten the time of sample inspection window for robustness test.

Variables	(1)	(2)	(3)	(4)
	PHS	PHS	DEP	DEP
GRE	−0.0183***	−0.0184***	−0.0613*	−0.0563**
	(−3.6849)	(−3.5844)	(−1.9249)	(−2.0230)
Individual controls	YES	YES	YES	YES
Family controls	NO	YES	NO	YES
_cons	0.0729	0.1715	13.0092***	13.3966***
	(0.3409)	(0.6181)	(22.3469)	(19.2282)
*N*	11,982	11,968	11,703	11,692
*R* ^2^	0.0959	0.1004	0.1314	0.1353

*T* value is indicated in brackets. *, **, *** represent the significance levels of 10%, 5% and 1%, respectively.

#### 5.2.4. Data trimming and addition of control variables

In order to test the reliability of the impact of urban green space on the health of middle-aged and older adults, tail-shrinking continuous variables. That is, the continuous variable was simplified by 1% bilateral contraction. The results are shown in Table 6. The regression coefficients were all significantly negative. In addition, because of the macro factors of the city, it may also have a certain impact on the health of residents. Here, this paper returns after examining the factors of urban meteorology, economy, medical care and industrial structure. Specifically, climate characteristic is measured by average temperature, urban economic development level is measured by logarithm of urban real GDP, medical level is characterized by logarithm of urban beds, and industrial structure is measured by the proportion of the added value of tertiary industry to the added value of secondary industry. The regression results are shown in columns (5) and (6), and the results are still robust after adding macro variables.

**Table 6:** Data trimming and addition of control variables for robustness test.

Variables	(1)	(2)	(3)	(4)	(5)	(6)
	PHS	PHS	DEP	DEP	PHS	DEP
GRE	−0.0196***	−0.0205***	−0.0809***	−0.0765***	−0.0193*	−0.0631**
	(−3.4933)	(−3.3769)	(−2.7390)	(−2.8841)	(−1.7133)	(−1.9812)
Individual controls	YES	YES	YES	YES	YES	YES
Family controls	NO	YES	NO	YES	YES	YES
_cons	−1.0474***	−0.9694***	12.5202***	12.7560***	−1.4895***	15.1240***
	(−6.1536)	(−4.6452)	(23.8374)	(20.1010)	(−3.5362)	(7.4091)
*N*	25,256	25,210	24,932	24,892	24,976	24,654
*R* ^2^	0.1240	0.1278	0.1392	0.1418	0.1353	0.1406

*T* value is indicated in brackets. *, **, *** represent the significance levels of 10%, 5% and 1%, respectively.

### 5.3. Heterogeneity test

This study is based on the differences in respondents’ gender, age, marital status, education level, residence (urban or rural), geographical location (eastern, central, and western regions, north and south), and park distribution (62) to test the heterogeneity of group regression.

#### 5.3.1. Heterogeneity effect across gender

According to the gender differences of respondents, the heterogeneous effects of male and female respondents affected by urban green space were tested, respectively. The results in columns (1)–(4) in Table 7 respectively report the influence coefficient of per capita green space area on the physical illness and depression of male and female samples. It is observed that the improvement of per capita green space area on females physical illness is slightly better than that of males, and the increase of per capita green space area can improve the enthusiasm for male outdoor activities or physical exercise, thus improving the physiological health effect brought by per capita green space area. However, the per capita green area is more significant for female to improve their depression, which may be due to the influence of China’s traditional lifestyle of “males are outside, and females are inside.” Females have relatively sufficient time for leisure and entertainment, and the external environment, such as green parks, easily influences their mood and mental health. Therefore, the per capita green area has a more significant mental health effect.

**Table 7:** The result of based on gender heterogeneity.

Variables	(1)	(2)	(3)	(4)
	PHS	DEP	PHS	DEP
	Male		Female	
GRE	−0.0208***	−0.0528**	−0.0134***	−0.0684**
	(−3.0029)	(−2.3552)	(−2.8322)	(−2.5526)
Individual controls	YES	YES	YES	YES
Family controls	YES	YES	YES	YES
_cons	−1.1175***	10.8578***	−0.6968***	15.5867***
	(−4.7525)	(16.0437)	(−2.9095)	(20.4314)
*N*	12,147	12,021	13,063	12,871
*R* ^2^	0.1156	0.0926	0.1330	0.1319

*T* value is indicated in brackets. *, **, *** represent the significance levels of 10%, 5% and 1%, respectively.

#### 5.3.2. Heterogeneity effect across age

According to the heterogeneity of age, the respondents were divided into two sample groups: those over 60 and those under 60. Columns (1)–(4) of Table 8 report the results of group regression based on age heterogeneity: urban green space area has a more apparent inhibitory effect on physical diseases and depression of the older adults over 60 years old. The possible reason is that, on the one hand, research shows that older adults are more sensitive to external environmental health than younger people (63). On the other hand, due to retirement and other reasons, the older adults will increase their outdoor leisure time, which will positively promote their health, improve their social participation, increase social support, and improve cohesion of the community. Therefore, the per capita green space area has more pronounced effects on the physical health and mental health of the older adults over 60 years old (64). The working hours of middle-aged people under the age of 60 occupy a more significant part of their lives. At this time, the marginal inhibitory effect of urban green space on physical diseases and depression is significantly reduced.

**Table 8:** The result of based on age heterogeneity.

Variables	(1)	(2)	(3)	(4)
	PHS	DEP	PHS	DEP
	>=60		<60	
GRE	−0.0238***	−0.0639**	−0.0066	−0.0593**
	(−5.4167)	(−2.5494)	(−1.2157)	(−2.4677)
Individual controls	YES	YES	YES	YES
Family controls	YES	YES	YES	YES
_cons	1.6027***	11.6442***	1.2468***	12.7072***
	(8.4357)	(18.5822)	(7.7511)	(18.2035)
*N*	13,373	13,122	11,904	11,836
*R* ^2^	0.0769	0.1324	0.0635	0.1460

*T* value is indicated in brackets. *, **, *** represent the significance levels of 10%, 5% and 1%, respectively.

#### 5.3.3. Heterogeneity effect across education level

According to their actual years of education, the interviewees are divided into those who have completed 9-year compulsory education and those who have not. The results in Table 9 show that the per capita green space area is somewhat influenced by the heterogeneity of education level. By observing the regression coefficient, we know that urban green space can significantly reduce the physical illness and depression of the older adults regardless of their low education level or high education level, and it significantly promotes physical health and mental health. However, this positive impact is more significant in the samples with high education levels. Some scholars believe that the difference in education level is the root cause of the health gap, and education can improve health by improving an individual’s internal function and living ability, which helps develop social and psychological resources (65).

**Table 9:** The result of based on the heterogeneity of education level.

Variables	(1)	(2)	(3)	(4)
	PHS	DEP	PHS	DEP
	Higher education level		Low education level	
GRE	−0.0192***	−0.0727***	−0.0149**	−0.0672**
	(−3.4353)	(−4.0712)	(−2.3425)	(−2.2619)
Individual controls	YES	YES	YES	YES
Family controls	YES	YES	YES	YES
_cons	−1.0453***	11.3259***	−0.8897***	10.4367***
	(−3.5194)	(13.2329)	(−4.5434)	(15.6741)
*N*	7,865	7,818	17,345	17,074
*R* ^2^	0.1197	0.1153	0.1338	0.1214

*T* value is indicated in brackets. *, **, *** represent the significance levels of 10%, 5% and 1%, respectively.

#### 5.3.4. Heterogeneity effect across marital status

According to the marital status of the respondents, they are divided into married and non-married. Columns (1)–(4) of Table 10 report the results of heterogeneous regression analysis based on marital status. For married people, the positive effect of urban per capita green space areas on psychological and physical health is more apparent, and the effect on physical illness and depression is significantly harmful. However, it does not significantly affect middle-aged and older adults in non-married status. The possible reason is that married or cohabiting middle-aged and older adults are more willing to increase their outdoor entertainment time with the company of their families. A stable and secure relationship is very important (66), and at the same time, their health status will be significantly improved. In contrast, middle-aged and older adults in non-married status feel more lonely because of the lack of family companionship, and negative emotions cannot be resolved in time under the comfort of family members (67). This has caused long-term depression, and it is difficult to have a positive impact through the per capita green space area of the city.

**Table 10:** The result of based on the heterogeneity of marital status.

Variables	(1)	(2)	(3)	(4)
	PHS	DEP	PHS	DEP
	Married		Non-married	
GRE	−0.0193***	−0.0794***	−0.0032	0.0530**
	(−3.6483)	(−3.2453)	(−0.4262)	(2.0959)
Individual controls	YES	YES	YES	YES
Family controls	YES	YES	YES	YES
_cons	−1.1432***	10.5319***	0.3118	15.9082***
	(−5.5485)	(18.5565)	(0.7744)	(10.4010)
*N*	21,956	21,730	3,254	3,162
*R* ^2^	0.1302	0.1346	0.0984	0.1172

*T* value is indicated in brackets. *, **, *** represent the significance levels of 10%, 5% and 1%, respectively.

#### 5.3.5. Heterogeneity effect across urban and rural areas

According to the urban-rural classification variables provided by CHARLS, based on the National Bureau of Statistics data, all the respondents were divided into urban areas and rural areas for sub-sample regression. Columns (1)–(4) in Table 11 report the results of the grouping test based on the heterogeneity of urban and rural areas. The results show that the effect of green space in rural areas on reducing physical diseases and emotional depression of the older adults is higher than that in urban areas. For this result, we give a reasonable explanation: each city’s number of parks and green spaces per capita is minimal. At the same time, due to the rapid development of urbanization, the enjoyment of green spaces by urban residents has been severely squeezed, and the developed transportation system has brought severe environmental pollution problems, resulting in the availability of barrier-free green spaces such as parks and forests in urban areas is often worse than that in rural areas (68). Therefore, the positive effect of green space on improving the physical and mental health of the older adults is more significant in rural areas.

**Table 11:** The result of based on the heterogeneity of urban and rural areas.

Variables	(1)	(2)	(3)	(4)
	PHS	DEP	PHS	DEP
	Rural		Uran	
GRE	−0.0216*	−0.1226***	−0.0143***	−0.0264**
	(−1.9082)	(−3.0845)	(−3.6951)	(−2.0620)
Individual controls	YES	YES	YES	YES
Family controls	YES	YES	YES	YES
_cons	−0.6611***	12.5992***	−1.3853***	13.3708***
	(−2.6465)	(16.9657)	(−5.1093)	(16.4081)
*N*	14,968	14,770	10,242	10,122
*R* ^2^	0.1162	0.1372	0.1433	0.1165

*T* value is indicated in brackets. *, **, *** represent the significance levels of 10%, 5% and 1%, respectively.

#### 5.3.6. Heterogeneity effect across regional

According to the economic development of the interviewee’s region, the interviewee’s region is divided into the eastern, central, and western regions for heterogeneity test. Table 12 (1)–(6) reports the results of the heterogeneity test based on the grouping regression of the eastern, central, and western regions. It is found that urban green space has a more noticeable impact on the health of middle-aged and older adults in the eastern region, and can significantly alleviate the physical illness and depression of older adults in the eastern region. However, it has no significant impact on the health of middle-aged and older adults in the western and central regions. The possible reason is that the economic development level in the eastern region is higher than in the western and central regions, and the residents’ demand for urban greening has increased. The economically developed cities in China are more concentrated in the eastern coastal cities, and the acceleration of urbanization has caused “urban diseases” and air pollution (69), which is harmful to the physical and mental health of the older adults.

**Table 12:** The result of based on regional heterogeneity.

Variables	(1)	(2)	(3)	(4)	(5)	(6)
	PHS	DEP	PHS	DEP	PHS	DEP
	Eastern		Central		Western	
GRE	−0.0101*	−0.0324*	−0.0192	−0.0734	−0.0188	−0.0272
	(−1.9058)	(−1.7811)	(−1.0066)	(−1.3015)	(−0.9959)	(−0.4264)
Individual controls	YES	YES	YES	YES	YES	YES
Family controls	YES	YES	YES	YES	YES	YES
_cons	−1.0296***	12.0424***	−1.0656***	12.5742***	−0.3133	14.1821***
	(−3.8038)	(15.3862)	(−3.3140)	(12.3425)	(−0.8263)	(10.8848)
*N*	11,314	11,155	8,389	8,296	5,501	5,435
*R* ^2^	0.1339	0.1293	0.1148	0.1236	0.1295	0.1736

*T* value is indicated in brackets. *, **, *** represent the significance levels of 10%, 5% and 1%, respectively.

#### 5.3.7. Heterogeneity effect across north and south

Taking the Qinling-Huaihe River as the boundary, the respondents’ location is divided into the south and the north, and the results of the grouping regression heterogeneity test are reported in columns (1)–(4) of Table 13. It can be seen that the impact of urban green space on the health of middle-aged and older adults in the southern region is significantly higher than that in the northern region. The possible reason is that the air pollution level in the north is higher than that in the south, and the increase of urban green space area will significantly improve air quality and increase people’s outdoor travel time, thus improving people’s health.

**Table 13:** The result of based on the heterogeneity of north and south regions.

Variables	(1)	(2)	(3)	(4)
	PHS	DEP	PHS	DEP
	North		South	
GRE	−0.0119**	−0.0526***	−0.0322***	−0.1170**
	(−2.5992)	(−3.2677)	(−2.6493)	(−2.0839)
Individual controls	YES	YES	YES	YES
Family controls	YES	YES	YES	YES
_cons	−0.7898***	12.6221***	−0.7761**	11.1593***
	(−2.9091)	(18.2760)	(−2.2514)	(8.3931)
*N*	12,765	12,581	11,456	11,330
*R* ^2^	0.1294	0.1366	0.1343	0.1457

*T* value is indicated in brackets. *, **, *** represent the significance levels of 10%, 5% and 1%, respectively.

#### 5.3.8. Heterogeneity effect across park quantity distribution

Because the appeal study cannot fully reflect the influence of the heterogeneity of urban parks on the health of the older adults, this paper makes a heterogeneous group regression analysis based on the number of urban parks. Specifically, the average number of urban parks is obtained nationwide, and cities with more than or equal to the average number of urban parks are regarded as the group with more parks; on the contrary, cities with less than the average number of urban parks are regarded as the group with fewer parks. A more significant number of urban parks means that the distribution of urban parks is more dispersed, while a smaller number of parks means that the publication of urban parks is more concentrated. The results of heterogeneous regression are shown in columns (1)–(4) of Table 14. The results show that in the areas where the number of urban parks is less, that is, if the distribution of urban parks is more concentrated, urban green space can improve the physical illness and depression of the older adults better. The possible reason is that the distribution in urban parks is more concentrated, which to some extent reflects the larger scale of parks, perfect infrastructure, and good planning of park entertainment and fitness functions. The effect of air purification and noise isolation produced by green vegetation is more pronounced, and larger and more concentrated urban green spaces can have an excellent positive effect on the health of the older adults.

**Table 14:** The result of based on the heterogeneity of park quantity distribution.

Variables	(1)	(2)	(3)	(4)
	PHS	DEP	PHS	DEP
	More parks		Fewer parks	
GRE	−0.0030	−0.0349***	−0.0464***	−0.1591***
	(−0.4908)	(−4.1298)	(−3.1501)	(−2.9396)
Individual controls	YES	YES	YES	YES
Family controls	YES	YES	YES	YES
_cons	−1.1041***	13.0794***	−0.9388***	12.1258***
	(−3.1676)	(15.1814)	(−3.9900)	(16.8437)
*N*	6,144	6,036	19,066	18,856
*R* ^2^	0.1304	0.1153	0.1263	0.1450

*T* value is indicated in brackets. *, **, *** represent the significance levels of 10%, 5% and 1%, respectively.

## 6. Mechanism test

There are few pieces of literature about the channels through which air pollution affects the health of middle-aged and older adults. Based on the mechanism discussion above, the micro-mechanism of the impact of urban green space on the health of middle-aged and older adults will be analyzed from the changes in extremely high-temperature weather and air quality. The annual extreme highest temperature of Celsius is used as an index to measure high-temperature weather, and the annual average value of PM_2.5_ is used as an index to measure air quality to measure the impact mechanism of urban green space on the health of middle-aged and older adults. The results are shown in Table 15. It is found that the increase of urban green space area significantly reduces the extreme maximum temperature and air pollution level. Specifically, for every square meter of green park space per capita, the annual extreme highest temperature decreases by 0.0694°C, and the annual average value of PM_2.5_ decreases by 0.5236 because urban green space helps to reduce the surface temperature and the heat island effect. This can prevent residents from being in a high-temperature environment for a long time and reduce the risk of heatstroke and other diseases related to high temperature. In addition, urban green space can effectively absorb pollutants in the air, reduce suspended particles in the air and improve air quality, which is conducive to reducing the risk of respiratory diseases for residents. At the same time, green plants have a calming effect on people’s psychology so that the central nervous system can easily adjust and improve the body’s function, giving people a feeling of tranquility, comfort, vitality, and spirit, thus improving their mental health. Moreover, increasing urban green space areas can further alleviate people’s nervous and depressed moods, and reduce the secretion of adrenaline and the excitability of human sympathetic nerves to a certain extent, thus reducing people’s mental stress. To sum up, the study found that the positive impact of urban green space on the health of middle-aged and older adults is mainly conducted by reducing the annual highest temperature and the annual average value of PM_2.5_, which is consistent with the hypothesis of the mechanism discussion mentioned above.

**Table 15:** The result of mechanism test.

Variables	(1)	(2)	(3)	(4)
	TEM	TEM	PM_2.5_	PM_2.5_
GRE	−0.0803***	−0.0694***	−0.5789***	−0.5236***
	(−36.6208)	(−29.6039)	(−23.1685)	(−19.8967)
Individual controls	NO	YES	NO	YES
Family controls	NO	YES	NO	YES
_cons	38.3367***	38.3523***	49.7061***	54.8864***
	(3351.3551)	(399.2156)	(381.5433)	(50.8612)
*N*	34,041	25,210	34,041	25,210
*R* ^2^	0.0379	0.0516	0.0155	0.0405

*T* value is indicated in brackets. *, **, *** represent the significance levels of 10%, 5% and 1%, respectively.

## 7. Discussion

Based on the incidence data of acute and chronic diseases and the depression scale of the older adults in the China Health and Retirement Longitudinal Study (CHARLS), this study considered the health status of the older adults in China from physical and psychological perspectives. On this basis, matching the data of urban per capita green space areas, this paper discusses the influence of urban per capita green space areas on the physical illness and depression of the older adults. It tests the mechanism path behind the influence of urban per capita green space areas on the physical and mental health of the older adults. The results show that the increase of urban per capita green areas can significantly improve the physical and mental health of the older adults. Compared with the previous research results, it is found that some studies believe that contacting with nature has a positive impact on people’s health, and the differences in the nature, size, distance, and quality of urban green space are discussed (70). Exposure to green space is associated with reducing people’s stress, depression, and anxiety symptoms and increasing happiness (71). The areas and samples investigated by researchers are different, and the methods of investigation and research are also different. However, they all affirm the conclusion that urban green space can improve people’s health well. Scholars’ analysis of different samples shows the diverse functions of urban green spaces with different properties (72). Studying spatial differences gives us a deeper understanding of this scientific problem. This study focuses on the interaction between urban green space and the older adults and can fully understand the spatial distribution heterogeneity of per capita green space in China, providing a reference for future research and practice.

Based on verifying the health effect of urban green space, this study further explored the innovative mechanism path. High-temperature weather and air quality are the necessary mechanisms for urban green space to affect the health of middle-aged and older adults because urban green space helps to reduce the surface temperature and alleviate the heat island effect (39, 73). This can prevent residents from being exposed to high temperatures for a long time and reduce the risk of heatstroke and other diseases related to high temperatures. In addition, urban green space can effectively absorb pollutants in the air, reduce suspended particles in the air, improve air quality, and help reduce the risk of diseases among residents (74). This inspires us to help residents suffering from urban heat island effects and air pollution and provides an operable mechanism for improving the health status of sub-healthy people.

Although this study clarifies the relationship between urban per capita green space area and physical and mental health of the older adults, it is inevitable that there are some limitations. First of all, there are some limitations in the use of health data in this study. We have not obtained the health data of the older adults after 2018. It may not be comprehensive enough to study the influencing mechanism path only from air pollution and urban extreme temperature. In the future, we will further test the healthier effects of urban green space, such as social contact and social cohesion; Secondly, the impact of urban green space on improving the health status of the older adults may show a dynamic feature in stages with the progress of time. Future research can distinguish the long-term and short-term effects of urban green space on health and conduct discussion and research in stages. Finally, the number of per capita areas may be difficult to reflect the impact of the spatial distribution of urban green space on people’s health. In the future, we will consider using spatial analysis method to accurately locate the distribution of urban parks and do further research, which will also become one of the schemes to optimize the quality of urban green space. Moreover, combining provincial, municipal and county-level data, and discussing in depth the influence on the health level of the middle-aged and older adults from a macro perspective, which is one of the future research focuses.

## 8. Conclusion

This study used data from the China Health and Retirement Longitudinal Study (CHARLS) in 2013, 2015, and 2018 to explore the causal relationship between urban green space and the health of middle-aged and older adults. The study found that increasing urban green space areas can significantly improve the physical and mental health of middle-aged and older adults, and the robustness test confirmed the above conclusions. Heterogeneous regression analysis was conducted on the gender, age, marital status, education level, residence (urban and rural), geographical location (eastern, central, western, northern and southern regions) and park quantity distribution. It can be found that the increase of urban green space areas has a more significant impact on the health of male groups, older groups, married or cohabiting groups, and middle-aged and older adults living in rural areas, areas with high economic development levels (eastern and central regions), northern regions and parks are more concentrated. In addition, further research found that high-temperature weather and air quality are essential mechanisms for urban green space to affect the health of middle-aged and older adults, and urban green space can improve the health level of middle-aged and older adults by reducing the temperature value of extremely high-temperature weather and improving air quality.

## Data availability statement

The datasets presented in this study can be found in online repositories. The names of the repository/repositories and accession number(s) can be found at: (1) Micro-individual data in this paper come from 2013, 2015, and 2018 follow-up surveys of China Health and Retirement Longitudinal Study (CHARLS data source: https://charls.charlsdata.com/); (2) The data of urban green space, air quality, and high-temperature weather from 2013 to 2018 in the yearbook for analysis and according to the city code and year, the Statistical Yearbook of Urban Construction (Data source: https://data.cnki.net/yearBook/single? Id = N2023010064) China Environmental Statistics Yearbook (Source: https://data.cnki.net/yearBook/single? Id = N2022030234).

## Author contributions

QL, YL, and LY: conceptualization and project administration. QL and JG: methodology. YL and XC: software and data curation. JG and XZ: validation. QL: writing—original draft preparation and funding acquisition. YL, LY, XC, and XZ: writing—review and editing. QL and XC: visualization. QL, YL, and XC: supervision. All authors contributed to the article and approved the submitted version.
